# Heterochronic parabiosis alters the transcriptomic landscape to combat aging and aging-related diseases in aging-accelerated mice

**DOI:** 10.1093/lifemedi/lnaf025

**Published:** 2025-07-01

**Authors:** Haochen Wang, Wencong Lyu, Yuzhe Sun, Xinyi Jia, Jinlong Bi, Ran Wei, Zhehao Du, Fanju Meng, Jianuo He, Shiyi Wang, Lijun Zhang, Chao Nie, Wei Tao

**Affiliations:** The MOE Key Laboratory of Cell Proliferation and Differentiation, School of Life Sciences, Peking University, Beijing 100871, China; The MOE Key Laboratory of Cell Proliferation and Differentiation, School of Life Sciences, Peking University, Beijing 100871, China; BGI Research, Beijing 102601, China; The MOE Key Laboratory of Cell Proliferation and Differentiation, School of Life Sciences, Peking University, Beijing 100871, China; The MOE Key Laboratory of Cell Proliferation and Differentiation, School of Life Sciences, Peking University, Beijing 100871, China; The MOE Key Laboratory of Cell Proliferation and Differentiation, School of Life Sciences, Peking University, Beijing 100871, China; The MOE Key Laboratory of Cell Proliferation and Differentiation, School of Life Sciences, Peking University, Beijing 100871, China; The MOE Key Laboratory of Cell Proliferation and Differentiation, School of Life Sciences, Peking University, Beijing 100871, China; The MOE Key Laboratory of Cell Proliferation and Differentiation, School of Life Sciences, Peking University, Beijing 100871, China; The MOE Key Laboratory of Cell Proliferation and Differentiation, School of Life Sciences, Peking University, Beijing 100871, China; The MOE Key Laboratory of Cell Proliferation and Differentiation, School of Life Sciences, Peking University, Beijing 100871, China; BGI Research, Shenzhen 518083, China; The MOE Key Laboratory of Cell Proliferation and Differentiation, School of Life Sciences, Peking University, Beijing 100871, China

**Keywords:** heterochronic parabiosis, snRNA-seq, aging, aging-related diseases

## Abstract

Aging is a multifactorial process involving a gradual decline in cellular and tissue functions, making it a major risk factor for aging-related degenerative diseases. In this study, we utilized the senescence-accelerated mouse prone 8 mice model, which mimics pathological characteristics of Alzheimer’s disease, fatty liver disease, and cardiac fibrosis, to construct a heterochronic parabiosis model and systematically investigate the rejuvenating effects of heterochronic parabiosis on the brain, liver, and heart. Our findings revealed that heterochronic parabiosis promotes synaptic plasticity and neuronal communication, restores hepatocyte metabolic functions, and reduces chronic inflammation and fibrosis in the heart. Notably, heterochronic parabiosis significantly downregulates the expression of age-related disease risk genes. In addition, endothelial cells, as cell types directly exposed to the circulatory environment, demonstrated the highest sensitivity to heterochronic parabiosis across three organs, and exhibited significantly reduced inflammation after intervention, suggesting that they may play an early and central role in the rejuvenation process. Overall, our study increases the understanding of the molecular and cellular mechanisms of aging and its related diseases, highlights the multiorgan and multitarget potential of heterochronic parabiosis in delaying aging and mitigating aging-related diseases, and provides new therapeutic targets for achieving healthy aging.

## Introduction

Aging is a complex biological process characterized by a progressive decline in physiological function, increased susceptibility to diseases, and a diminished capacity to maintain homeostasis [1, 2]. As a major risk factor for a wide array of chronic conditions, such as neurodegenerative disorders, cardiovascular diseases, metabolic syndromes, and organ fibrosis [3–5]. Age-related diseases arise from the gradual buildup of molecular and cellular damage, leading to inflammation, metabolic imbalance, and reduced tissue regeneration [3, 6–9]. The characteristics and mechanisms of aging-related diseases, as well as their relationships with the aging process, warrant further investigation [2]. A deeper understanding of the mechanisms driving aging and its associated diseases is essential for developing effective therapeutic strategies aimed at promoting healthy aging.

One of the most effective methods for improving the function of aging tissues is heterochronic parabiosis [10], the surgical union of the circulatory systems of young and old animals. This technique has emerged as a powerful tool for studying the systemic factors that influence aging and rejuvenation. By connecting the circulatory systems of young and old animals, heterochronic parabiosis offers a unique platform for investigating how the systemic factors of young animals can ameliorate age-related tissue dysfunction and disease phenotypes in older animals [11, 12]. Previous studies have shown that heterochronic parabiosis can alleviate various pathologies associated with aging [13–15]. These findings underscore the potential of rejuvenation therapies targeting systemic factors as promising strategies for delaying aging and alleviating age-related diseases [16].

To investigate the pathological mechanisms of aging and aging-related diseases, the senescence-accelerated mouse prone 8 (SAMP8) line has been widely utilized [17, 18]. SAMP8 mice, characterized by accelerated aging and a shortened lifespan, present hallmark features of aging, including cognitive decline, chronic inflammation, metabolic abnormalities, and organ-specific degenerative changes [19]. SAMP8 mice share many features with human aging-related diseases, making them a suitable model for studying aging and its related disorders, such as Alzheimer’s disease (AD) [20], fatty liver disease [21], nonalcoholic steatohepatitis [22], and cardiac fibrosis [23]. The senescence-accelerated mouse resistance 1 (SAMR1) line, the antiaging counterpart of the SAMP8 line, served as a “young” control in these studies [24]. Comparative analyses between SAMP8 and SAMR1 mice offer valuable insights into the mechanisms underlying pathological aging and its associated diseases.

In this study, we established a SAMP8–SAMR1 heterochronic parabiosis mouse model and employed single-nucleus transcriptomics to elucidate the molecular and cellular mechanisms underlying the rejuvenation effects induced by heterochronic parabiosis. Focusing on three key organs (brain, liver, and heart), we identified rejuvenation-associated pathways and investigated how the circulatory environment of young mice facilitates the rejuvenation of aging tissues in aging mice. Our findings demonstrate that heterochronic parabiosis not only abrogates age-related characteristics in these organs but also downregulates disease-related risk genes, effectively combating both aging and aging-associated diseases. These results provide new insights into how heterochronic parabiosis modulates aging-related disease pathways and offers potential therapeutic targets for mitigating the detrimental effects of aging.

## Results

### Heterochronic parabiosis single-nucleus transcriptomic profiling

**Figure 1.**
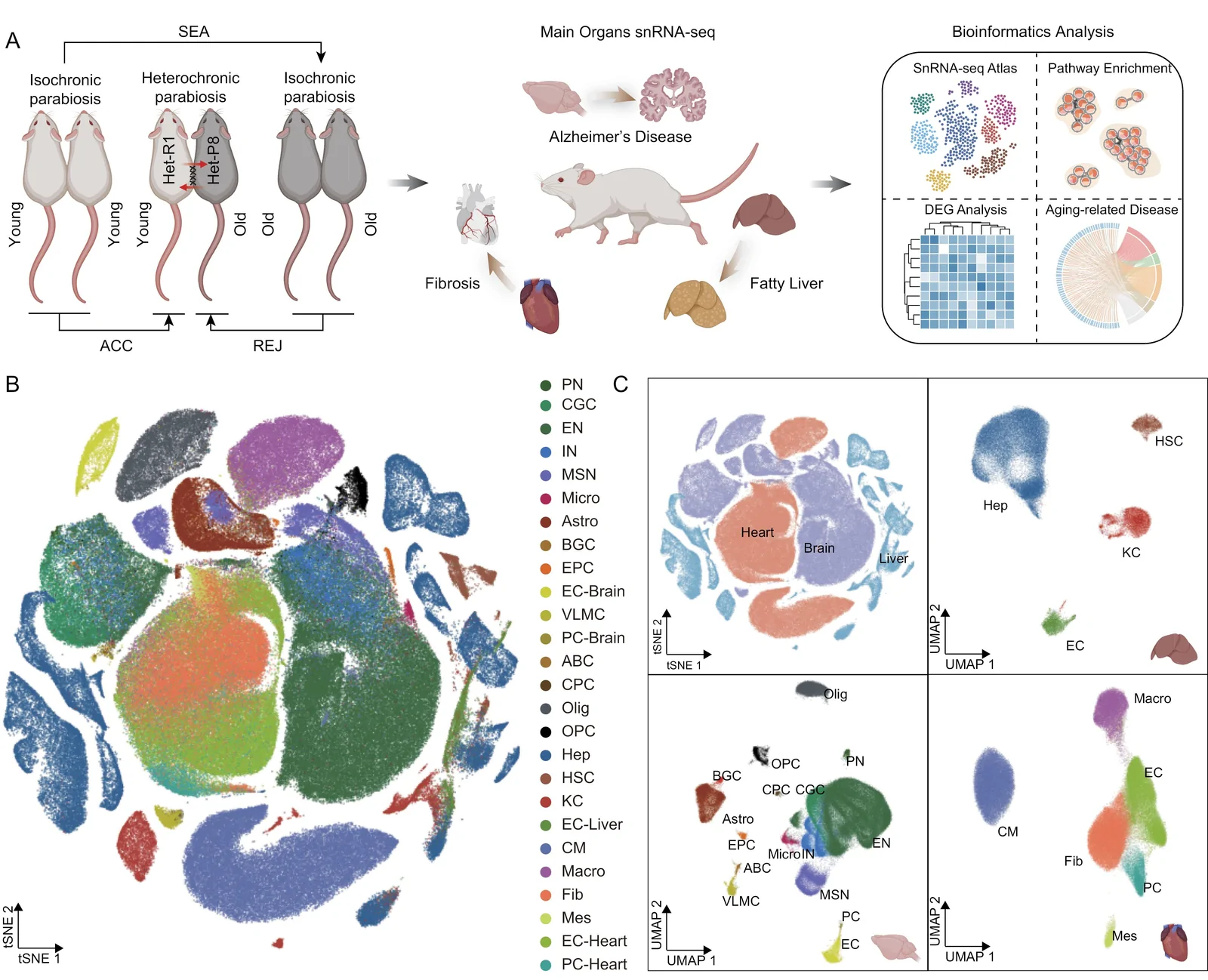
Construction of a single-nucleus transcriptomic atlas of heterochronic parabiosis across the brain, liver, and heart in mice. (A) Schematic diagram of isochronic or heterochronic parabiotic pairing and snRNA-seq. The left panel shows the experimental design, whereas the middle panel illustrates the three aging-related diseases (Alzheimer’s disease, fatty liver, and heart fibrosis) in the mice and the sampled organs (brain, liver, and heart). Iso-R1, isochronic parabiotic SAMR1; Iso-P8, isochronic parabiotic SAMP8; Het-R1, heterochronic parabiotic SAMR1; Het-P8, heterochronic parabiotic SAMP8. SEA, senescence acceleration; REJ, rejuvenation; ACC, acceleration. (B) t-SNE plot showing different cell types across three organs on the basis of snRNA-seq data. PN, Purkinje neuron; CGC, cerebellar granule cell; EN, excitatory neuron; IN, inhibitory neuron; MSN, medium spiny neuron; Micro, microglia; Astro, astrocyte; BGC, Bergmann glial cell; EPC, ependymocyte; EC-Brain, endothelial cell in the brain; VLMC, vascular leptomeningeal myoendothelial cell; PC-Brain, pericyte in the brain; ABC, arachnoid barrier cell; CPC, choroid plexus epithelial cell; Olig, oligodendrocyte; OPC, oligodendrocyte precursor cell; Hep, hepatocyte; HSC, hepatic stellate cell; KC, Kupffer cell; EC-liver, endothelial cell in the liver; CM, cardiomyocyte; Macro, macrophage; Fib, fibroblast; Mes, mesothelial cell; EC-Hearts, endothelial cell in the heart; PC-Heart, pericyte in the heart. t-SNE, t-distributed stochastic neighbor embedding. (C) t-SNE or UMAP plots showing the cell composition across the brain, liver, and heart. UMAP, uniform manifold approximation and projection.

We performed high-throughput single-nucleus RNA sequencing (snRNA-seq) to obtain the transcriptional profiles of SAMR1 and SAMP8 mice after parabiosis (Fig. 1A). We surgically connected SAMP8 and SAMR1 mice as heterochronic pairs (Het-P8/Het-R1) following a previously reported method [25], and isochronic pairs between SAMR1 mice (Iso-R1) and SAMP8 (Iso-P8) were surgically prepared to serve as controls. All pairs were kept together for 4‒5 weeks prior to tissue collection and subsequent analysis.

To investigate the cellular mechanisms through which heterochronic parabiosis influences aging or aging-related diseases and its rejuvenation effects, we isolated and compared three key organs of mice that play critical roles in age-related degenerative diseases, namely, the brain, liver, and heart. After performing snRNA-seq and rigorous quality control, a total of 29,8675 high-quality cells were collected from 12 parabionts (four isochronic SAMR1, Iso-R1; four isochronic SAMP8, Iso-P8; two heterochronic SAMR1, Het-R1; and two heterochronic SAMP8, Het-P8). Among them, 122,072 cells were collected from the brain, 121,040 from the heart, and 55,563 from the liver.

By integrating heterochronic and isochronic sample data and incorporating previously reported mouse tissue and organ marker genes, we identified 26 distinct cell types, representing the major cell types across the three organs [13, 26, 27] (Fig. 1B and 1C). For each cell type, we utilized multiple cell-type-specific marker genes, as described in the literature, to accurately determine cell-type identity. In the brain, we identified five types of neurons: excitatory neurons (*Slc17a7*^+^, *Slc17a6*^+^); inhibitory neurons (*Gad1*^+^, *Gad2*^+^); medium spiny neurons (*Drd1*^+^, *Adarb2*^+^); Purkinje neurons (*Calb1*^+^, *Car8*^+^); and cerebellar granule cells (*Cbln1*^+^, *Cbln3*^+^). In addition, 11 types of nonneurons were identified: microglia (*C1qa*^*+*^, *C1qb*^*+*^); astrocytes (*Gja1*^+^, *Aqp4*^+^); Bergmann glial cells (*Gdf10*^+^); ependymocytes (*Foxj1*^+^); endothelial cells (ECs, *Cldn5*^+^); vascular leptomeningeal myoendothelial cells (*Slc6a13*^+^); pericytes (PCs, *Kcnj8*^+^, *Pdgfrb*^+^); arachnoid barrier cells (*Slc47a1*^+^); choroid plexus epithelial cells (*Ttr*^+^); oligodendrocytes (*Mag*^+^, *Mog*^+^, *Olig1*^+^); and oligodendrocyte precursor cells (*Pdgfra*^+^) [28, 29]. In the liver, we identified four main cell types, namely, hepatocytes (Hep, *Sds*^+^, *Sult2a8*^+^), hepatic stellate cells (*Dcn*^+^), Kupffer cells (*Csf1r*^+^, *Cd5l*^+^), and ECs (*Stab2*^+^, *Vwf*^+^) [30, 31]. In addition, cardiomyocytes (CMs, *Myh7*^+^, *Myl2*^+^), macrophages (*Ptprc*^+^, *Itgam*^+^), fibroblasts (Fib, *Pcdh9*^+^), mesothelial cells (*Msln*^+^), ECs (*Egfl7*^+^), and PCs (*F13a1*^+^, *Abcc9*^+^) were present in the heart [32, 33]. Overall, we initially constructed snRNA-seq profiles for the brains, livers, and hearts of heterochronic parabiosis model SAMP8 and SAMR1 mice.

### The global rejuvenating effects of heterochronic parabiosis at the organ and cell-type levels in SAMP8 mice

**Figure 2.**
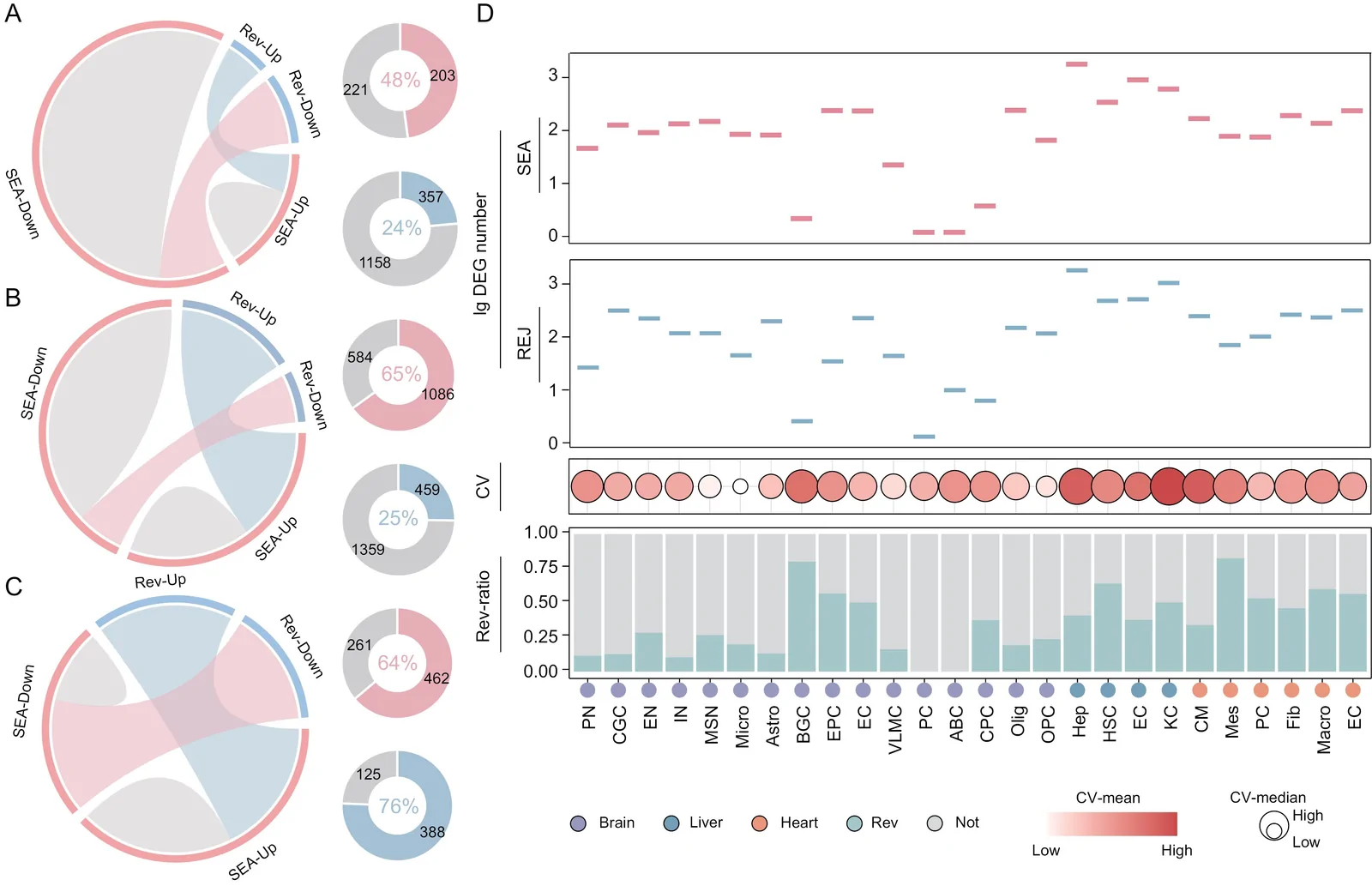
Overview of the rejuvenation effects of heterochronic parabiosis across multiple organs and cell types. (A) Chord diagram (left) and ring charts (right) showing the SEA upregulated (top) and downregulated (bottom) DEG reversed ratios in the brain. Rev-Up, SEA group upregulated DEG reversed ratio; Rev-Down, SEA group downregulated DEG reversed ratio. (B) Chord diagram (left) and ring charts (right) showing the SEA group upregulated (top) and downregulated (bottom) DEG reversed ratios in the liver. (C) Chord diagram (left) and ring charts (right) showing the SEA group upregulated (top) and downregulated (bottom) DEG reversed ratios in the heart. (D) Transcriptomic rejuvenation impact in each cell type. Lg DEG number, log10(DEG number); line plots showing the number of DEGs in the SEA and REJ groups. CV, coefficient of variation. Rev-Ratio, reverse gene ratio.

First, we assessed the rejuvenating effects of heterochronic parabiosis at the organ level (Fig. 2A–C). We first identified the differentially expressed genes (DEGs) between the SEA (Iso-P8/Iso-R1, senescence acceleration) and REJ (Het-P8/Iso-P8, rejuvenation) groups and then selected the genes whose expression patterns went in the reversed direction in the REJ group compared to the SEA group and calculated their expression ratios in the brain, liver, and heart between the two groups. A total of 1939 DEGs were identified in the brain, including 424 upregulated genes, of which 203 (48%) were reversed in the REJ group, and 1515 downregulated genes, of which 357 (24%) were reversed in the REJ group (Fig. 2A). A total of 3488 DEGs were identified in the liver, including 1670 upregulated genes, of which 1086 (65%) were reversed, and 1818 downregulated genes, of which 459 (25%) were reversed (Fig. 2B). A total of 1236 DEGs were identified in the heart, including 723 upregulated genes, of which 462 (64%) were reversed, and 513 downregulated genes, of which 388 (76%) were reversed (Fig. 2C). Collectively, these findings indicate that heterochronic parabiosis has more pronounced rejuvenating effects on the downregulated genes in the SEA group, which is consistent with previous reports [34].

Next, we explored the rejuvenating effects of heterochronic parabiosis at the cell-type level (Fig. 2D). The coefficient of variation (CV) is used to measure the variation in gene expression across different groups, reflecting in the degree of transcriptional noise [35, 36]. Here, we evaluated the rejuvenating effects of heterochronic parabiosis on gene expression at the cell-type level. This was done by comparing the CVs between the Iso-R1/Iso-P8 and Iso-R1/Het-P8 groups, with a larger CV difference indicating a more pronounced change. The Pearson correlation coefficient is commonly used to assess the relationship between the expression or transcriptomic features of genes in different samples [37]. We examined the similarities between the Iso-R1/Iso-P8 and Iso-R1/Het-P8 groups from the perspective of DEGs and explored which cell type presented the most changes from heterochronic parabiosis (Fig. S1). In general, at the cell-type level, Het-P8 mice show greater similarity to Iso-R1 mice, with the most pronounced rejuvenating effects observed in ECs in the brain, liver, and heart, as well as in CMs in the heart. Overall, we evaluated the impact of heterochronic parabiosis on various organs and cell types from multiple angles. While rejuvenating effects were observed across all tissues, the heart and liver presented more significant changes than the brain.

### Heterochronic parabiosis restores synaptic plasticity and neuron–neuron communication

**Figure 3.**
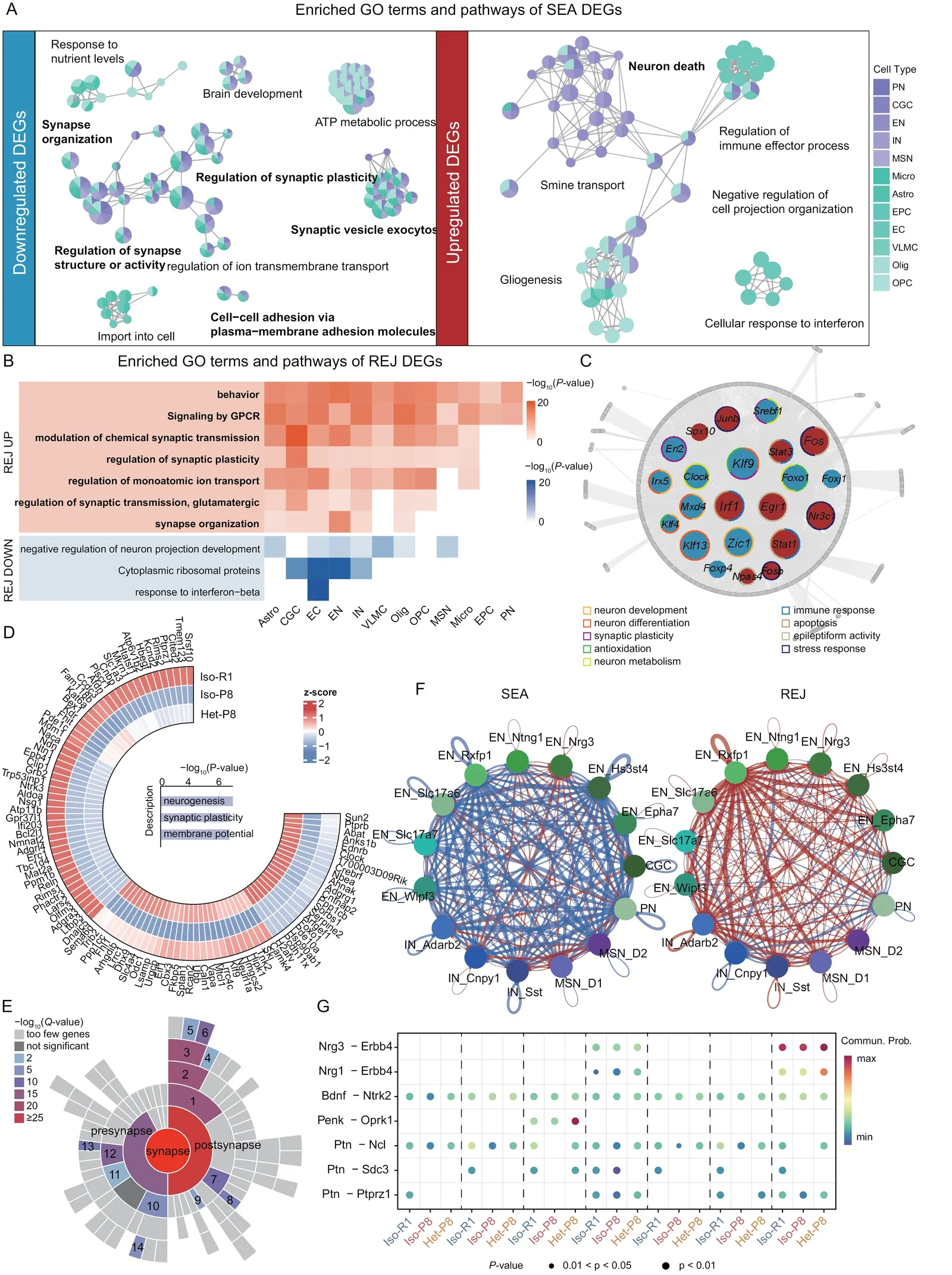
Promotion of synaptic plasticity and neuron-neuron communication by heterochronic parabiosis. (A) Network plot showing the enriched GO terms of downregulated (left) and upregulated (right) genes in the SEA group. (B) Heatmap showing the enriched GO terms of upregulated (top) and downregulated (bottom) genes in the REJ group across the main cell types. (C) Network plot showing the transcriptional regulators of SEA group DEGs. The node size reflects the target gene number. The inside nodes represent unregulated and downregulated transcriptional regulators. The outside nodes indicate the target genes regulated by corresponding transcriptional regulators. (D) Circle heatmap showing the abrogation of the downregulated expression of transcriptional regulators and target genes associated with neuron function. Middle bar plot showing the enriched GO terms of these genes. (E) Circular stacked plot showing enrichment of proteins localized to the synaptic compartment as annotated within the SynGO database within neuron REJ DEGs. 1, postsynaptic specialization. 2, postsynaptic density. 3, postsynaptic density membrane. 4, postsynaptic density, intracellular component. 4, postsynaptic density membrane. 5, extrinsic component of postsynaptic density membrane. 6, integral component of postsynaptic density membrane. 7, postsynaptic membrane. 8, integral component of postsynaptic membrane. 9, postsynaptic cytosol. 10, presynaptic active zone. 11, neuronal dense core vesicle. 12, presynaptic membrane. 13, integral component of presynaptic membrane. 14, integral component of presynaptic active zone membrane. (F) Summarization network plots showing the weights of ligand‒receptor interactions between neuron types in the SEA and REJ groups. Red indicates interaction upregulation, and blue indicates interaction downregulation. (G) Dot plot showing specific ligand‒receptor interaction heterochronic parabiosis reverse changes.

Compared with SAMR1 mice, SAMP8 mice exhibit many characteristics of neurodegenerative diseases [20, 38]. We confirmed this by performing pathway network analysis of the SEA DEGs, visualizing these changes at the single-cell nuclear transcriptomic level (Fig. 3A). Among the key genes that were downregulated in the SEA group, many are closely associated with neuronal function, and the enriched Gene Ontology (GO) terms included synapse organization, synaptic plasticity, synaptic vesicle exocytosis, and synapse structure or activity. In addition, the pathway of neuron death and regulation of the immune effector process were enriched in the upregulated genes. These results, which align with previously reported bulk RNA data from the brains of SAMP8 mice [39], also revealed impaired neurofunctional pathways at the cell-type level, highlighting age-related neurodegenerative features.

Heterochronic parabiosis can upregulate genes that were enriched in synaptic function-related pathways in SAMP8 mice (Fig. 3B), such as pathways associated with chemical synaptic transmission, synaptic plasticity, and synapse organization, indicating that heterochronic parabiosis may lead to the restoration of neuronal synaptic function by reactivating synaptic gene regulators. Next, to elucidate the synaptic transcriptional regulatory network, we used single-cell regulatory network inference and clustering (SCENIC) to predict the core transcription factors (TFs) that regulate the DEGs in the SEA group [40] (Fig. 3C). Among the TFs found to be dysregulated in the SEA group, *Klf9* is involved in controlling neuronal differentiation and synaptic plasticity and has been implicated in neuroprotection and the response to cellular stress [41, 42]. *Foxo1* regulates the oxidative stress response, and neuronal survival and is essential for maintaining neuronal health and function under stress conditions [43]. *Clock*, a core component of the circadian rhythm machinery, influences neuronal activity, and synaptic plasticity, thereby modulating cognitive functions and neurodegeneration [44, 45]. Moreover, *Klf9*, *Foxo1*, and *Clock*, which play significant roles in neurodegenerative diseases and age-related cognitive decline, regulate downstream genes associated with neurogenesis, synaptic plasticity, and membrane potential (Fig. 3D). More broadly, we annotated the genes whose expression was opposite between the groups using the SynGO database [46] and found that these genes play important roles in both presynaptic and postsynaptic functions, which are impaired in SEA (Figs. 3E and S2). These changes in gene expression indicate that heterochronic parabiosis may help restore neuronal synaptic function and potentially mitigate age-related neurodegeneration by modulating these key signaling pathways.

Synapses are key structures for information transmission between neurons [47, 48]. We performed a more detailed subcluster classification of the neurons (Fig. S3). Using CellChat, we found that heterochronic parabiosis significantly restored communication between neuron subtypes [49, 50] (Fig. 3F). We observed that heterochronic parabiosis can also restore certain key ligand–receptor interactions. The interaction between Nrg3/Nrg1 and Erbb4 is crucial for neurodevelopment and synaptic plasticity, helping to maintain neuroprotection [51]. Bdnf binding to Ntrk2 regulates neuron growth, survival, and synaptic plasticity, particularly by influencing learning and memory [52]. The interaction between Penk and Oprk1 receptors is involved in pain modulation, emotional control, and neuroprotection, affecting neuronal function [53]. Finally, the binding of Ptn with Ncl, Sdc3, and Ptprz1 plays a role in neuroregeneration and synaptic repair, supporting neuronal adhesion, signaling, and repair processes [54, 55] (Fig. 3G). Our computational analysis revealed a range of cell–cell communication networks involved in the regulation of neurodegeneration and synaptic dysfunction, which are disrupted during aging and subsequently modified by heterochronic parabiosis.

### Heterochronic parabiosis improves the metabolic functions of hepatocytes in various regions of the liver

**Figure 4.**
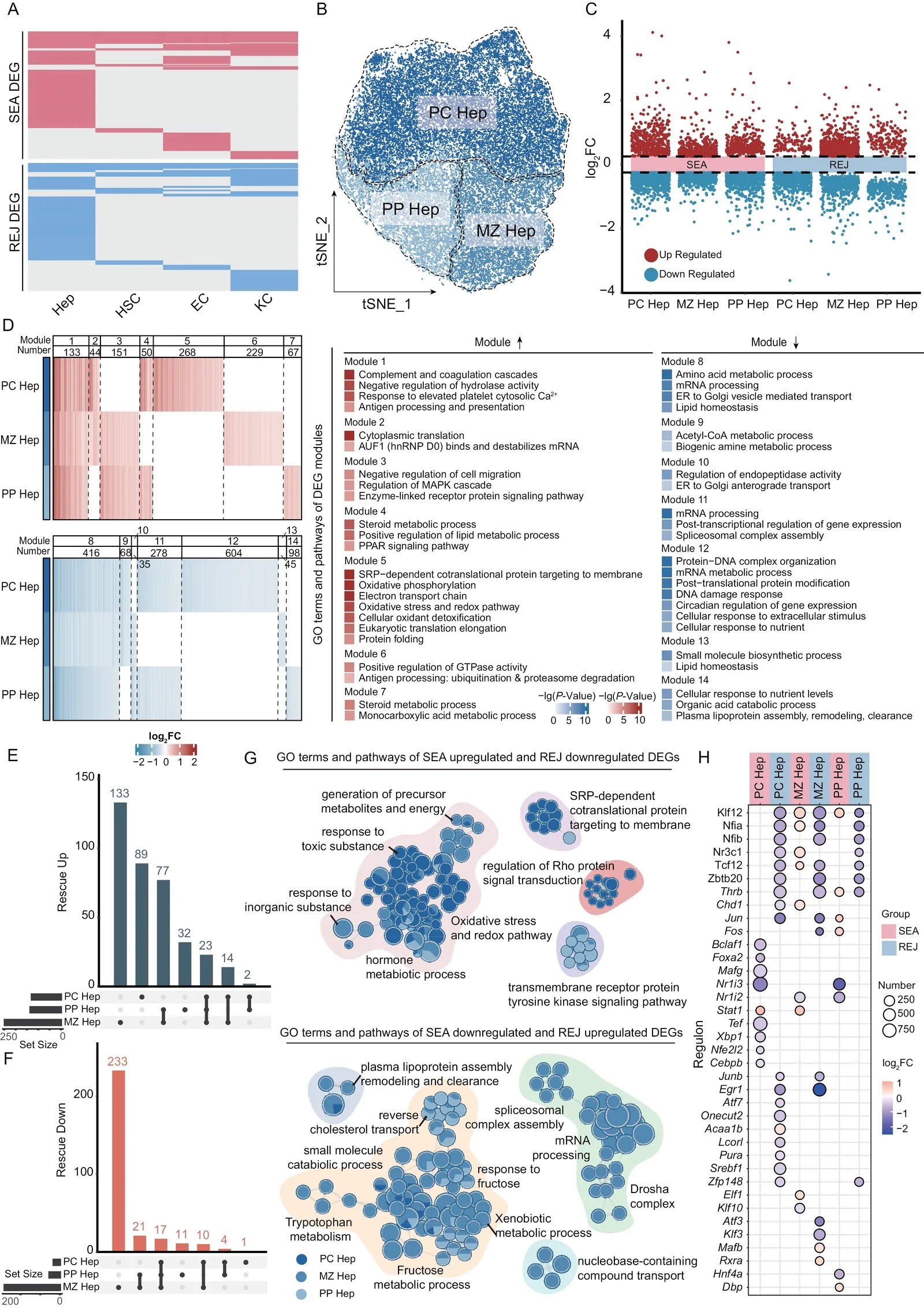
Restoration of the metabolic functions of hepatocytes in different hepatic regions by heterochronic parabiosis. (A) Heatmaps showing the distribution of DEGs in SEA and REJ liver cell groups. Genes were sorted according to their conservation and specificity across cell types. (B) t-SNE plot showing different subtypes of hepatocytes. PC Hep, pericentral hepatocyte; MZ Hep, middle zone hepatocyte; PP Hep, periportal hepatocytes. (C) Volcano plot showing DEGs across different hepatocyte subtypes in the SEA and REJ groups. (D) Heatmap (left) showing the 14 classified SEA group DEG modules according to the shared genes among subtypes. Heatmap (right) showing the enriched GO terms of the modules. (E) UpSet plot showing the number of rescued upregulated genes (shared genes between SEA group upregulated DEGs and REJ group downregulated DEGs) across the three hepatocyte subtypes. (F) UpSet plot showing the number of rescued downregulated genes (shared genes between SEA group downregulated DEGs and REJ group upregulated DEGs) across three hepatocyte subtypes. (G) Network plots showing the enriched GO terms of rescued upregulated and downregulated genes. The size of the dots reflects the number of genes in the pathway. (H) Dot plot showing the transcriptional regulators of DEGs across three hepatocyte subtypes in the SEA and REJ groups.

The livers of SAMP8 mice typically exhibit a decline in metabolic function, leading to increased fat accumulation and fatty liver disease, which are closely associated with metabolic pathway dysregulation during the aging process [22, 56], such as the suppression of the small molecule metabolic pathway and activation of the lipid metabolic pathway (Fig. S4A). We found that hepatocytes presented the greatest number of DEGs in both the SEA and REJ groups (Fig. 4A). The liver, with its distinct zones, performs different functions, and the aging process causes these zones to have distinct features [30, 36]. Therefore, we subdivided the hepatocytes into three groups on the basis of their location: pericentral hepatocytes (PC Hep), middle zone hepatocytes (MZ Hep), and periportal hepatocytes (PP Hep) (Figs. 4B and S4B), and identified the DEGs among the hepatocytes in each region (Fig. 4C).

To examine common and subtype-specific aging features in hepatocytes, we grouped SEA group DEGs into 14 modules based on shared gene expression across three hepatocyte subtypes (Fig. 4D). While many DEGs were unique to specific subtypes, downregulated genes commonly involved amino acid metabolism and lipid homeostasis, and upregulated genes were enriched in complement and coagulation pathways, indicating aging-related inflammation and metabolic imbalance. Each subtype showed distinct changes: SEA PC Hep cells had increased oxidative stress and reduced DNA damage response; MZ Hep cells showed enhanced GTPase activity and reduced small molecule biosynthesis; PP Hep cells showed increased steroid metabolism and decreased organic acid catabolism. Notably, the expression of Modules 7, 9, 13, and 14, related to metabolic processes, was significantly reversed in the REJ group (Fig. S4C).

To assess the impact of heterochronic parabiosis on different liver regions more accurately, we applied a more stringent criterion for identifying genes whose expression was restored after heterochronic parabiosis (Figs. 4E, 4F, and S4E). MZ Hep exhibited the greatest number of genes whose expression was restored and heterochronic parabiosis appeared to have a more pronounced rejuvenating effect on MZ Hep. This finding was further supported by principal component analysis (PCA) (Fig. S4D). MZ Hep plays a crucial role in restoring liver function and maintaining liver homeostasis [57]. We performed pathway enrichment analysis on these genes and revealed that heterochronic parabiosis can suppress the oxidative stress pathway and activate several metabolism-related pathways, such as the lipoprotein clearance pathway and the small molecule metabolic pathway (Figs. 4G and S4F). Moreover, through SCENIC TF analysis, we observed that heterochronic parabiosis is capable of stabilizing the expression of several metabolism-related TFs (Figs. 4H and S4G). *Klf12* and *Nfia* are important regulatory factors in fat synthesis and storage [58–60]. Furthermore, heterochronic parabiosis upregulated *Acaa1b*, which is involved in the oxidation of branched-chain fatty acids [61, 62]. In summary, heterochronic parabiosis can restore the expression of metabolism-related genes, decrease lipid accumulation by regulating TF expression, and alleviate aging-related metabolic dysregulation in SAMP8 mice across hepatocyte subtypes.

### Heterochronic parabiosis alleviates inflammation and fibrosis in the heart

**Figure 5.**
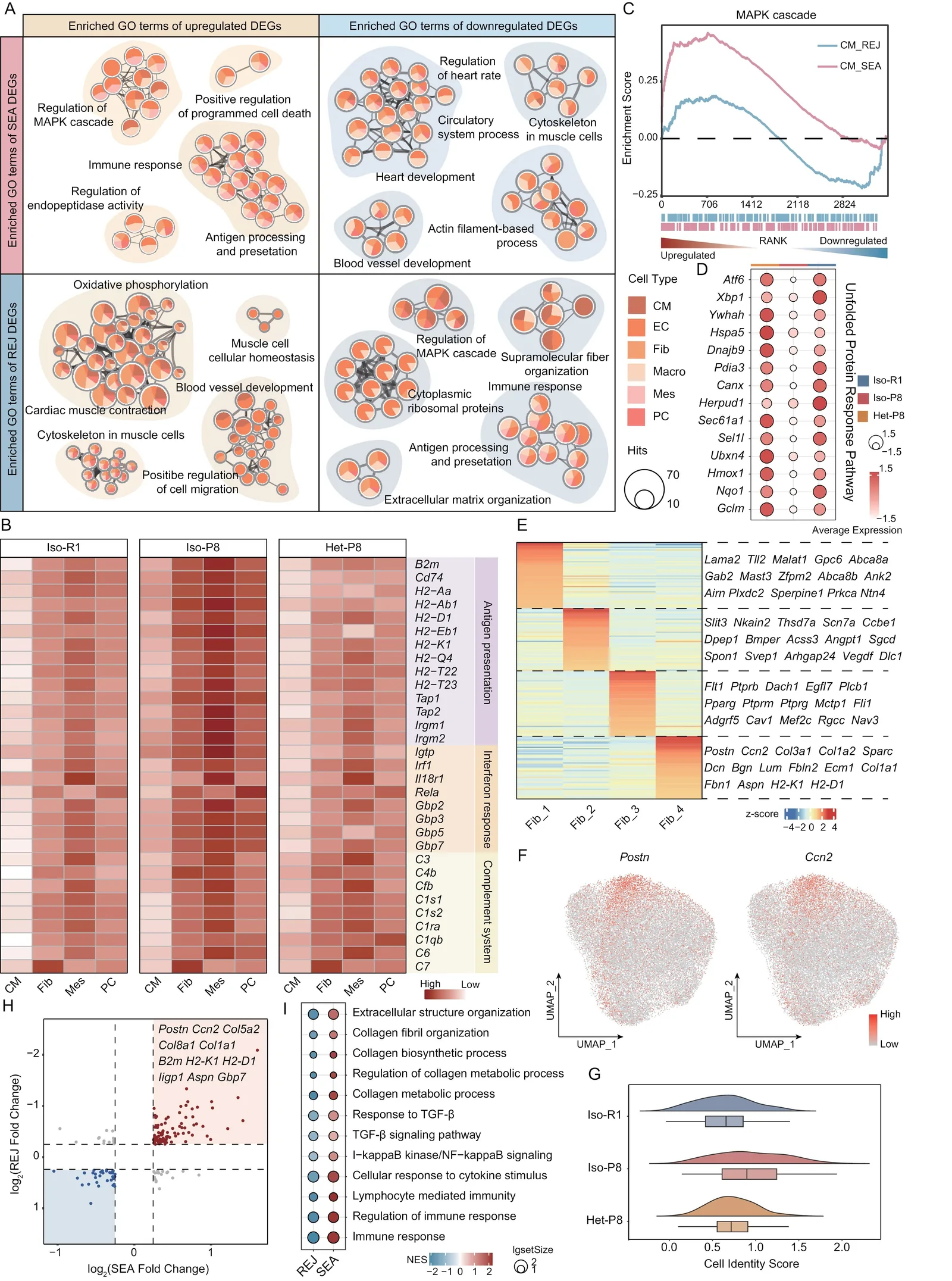
Alleviation of the inflammatory response and fibrosis in the heart by heterochronic parabiosis. (A) Network plots showing the enriched GO terms in the upregulated (left) and downregulated (right) DEGs of the SEA (top) and REJ (bottom) groups. (B) Heatmaps showing inflammatory-associated gene expression across heart main cell types in Iso-R1, Iso-P8, and Het-P8 mice. Fib, fibroblast, Mes, mesothelial cell, PC, pericyte. (C) GSEA plot showing MAPK cascade pathway changes in SEA and REJ cardiomyocytes. CM, Cardiomyocyte. GSEA, gene set enrichment analysis. (D) Dot plot showing key genes involved in the unfolded protein response pathway in Iso-R1, Iso-P8, and Het-P8 mice. (E) Heatmap showing the expression of fibroblast subtype markers derived from nonnegative matrix factorization (NMF). (F) UMAP plots showing *Postn* and *Ccn2* expression in Fib_4. (G) Violin and box plots showing the Fib_4 cell identity scores in Iso-R1, Iso-P8, and Het-P8 mice. (H) Dot plots showing the genes whose expression was rescued in Fib_4. Red represents the shared DEGs between SEA upregulated DEGs and REJ downregulated DEGs. Blue represents the shared DEGs between SEA group downregulated DEGs and REJ group upregulated DEGs. (I) Dot plot showing GSEA results of changes in fibrinogen synthesis and inflammation pathways in the DEGs of the SEA and REJ groups. NES, normalized enrichment score.

As aging progresses, the heart undergoes a series of structural and functional changes [63]. These changes are also observed in SAMP8 mice, which serve as an accelerated aging mouse model [23]. We first performed pathway enrichment analysis on the DEGs in the hearts of the SEA group mice and found that the pathways associated with programmed cell death and the immune response were enriched in the upregulated genes, which is consistent with reports of chronic inflammation in the hearts of SAMP8 mice [23] (Fig. 5A). In SAMP8 mice, downregulated DEGs were enriched in pathways related to heart development and muscle cytoskeleton, reflecting age-related cardiac degeneration. In contrast, REJ DEGs showed enrichment in pathways linked to cardiac muscle contraction and muscle cell homeostasis, indicating functional recovery (Fig. 5A). Heterochronic parabiosis also reduced heart inflammation by downregulating genes involved in antigen presentation (*B2m*, *H2-Aa*, *H2-D1*), interferon response (*Irf1*, *Gbp2*, *Gbp3*), and the complement system (*C3*, *C4b*, *C1s1*) (Fig. 5B). In addition, the MAPK pathway, previously activated in aging hearts, was significantly suppressed [23] (Fig. 5C). The MAPK signaling pathway plays a crucial role in stress responses, and during aging, dysregulation of the MAPK pathway may lead to chronic inflammation, fibrosis, and myocardial cell dysfunction [64]. We found that heterochronic parabiosis can restore the expression of the upstream TFs *Atf6* and *Xbp1* and downstream genes in the unfolded protein response caused by the MAPK pathway (Fig. 5D). In summary, heterochronic parabiosis can restore the homeostasis and function of cardiomyocytes in SAMP8 mice.

Cardiac fibrosis is caused by the activation of fibroblasts, which produce large amounts of collagen and other extracellular matrix (ECM) components. This leads to collagen deposition and fibrosis in cardiac tissue. GO enrichment analysis revealed that heterochronic parabiosis can suppress pathways related to ECM and fiber organization (Fig. 5A). To explore the potential molecular changes in fibroblasts during heterochronic parabiosis, we used nonnegative matrix factorization to analyze the distinct expression patterns of fibroblasts (Fig. 5E). We classified fibroblasts into four major subtypes on the basis of their characteristic marker expression (Fig. 5E). Fib_1 represents normal fibroblasts, whereas Fib_4 represents abnormally activated fibroblasts [65]. These activated fibroblasts express cardiac injury activation markers (*Postn*, *Ccn2*), as well as genes related to collagen synthesis (*Col1a2*, *Col3a1*) and inflammation-related genes (*H2-K1*, *H2-D1*) (Fig. 5F). Notably, the cell identity score of Fib_4 was increased in SAMP8 mice, whereas heterochronic parabiosis decreased this score (Fig. 5G). Thus, we identified Fib_4 SEA and REJ DEGs to further analyze the potential molecular mechanisms underlying the increase in the cell identity score of Fib_4 by heterochronic parabiosis (Fig. 5H). Heterochronic parabiosis restored the expression of Fib_4 markers, collagen synthesis genes, and inflammatory genes. GSEA showed that it suppressed the TGF-β pathway (fibrosis), NF-κB pathway (inflammation), and collagen synthesis pathways (Fig. 5I). These results suggest that heterochronic parabiosis alleviates cardiac fibrosis by reprogramming activated fibroblasts and modulating fibrosis- and inflammation-related signaling.

### Heterochronic parabiosis attenuates EC inflammation across multiple organs

**Figure 6.**
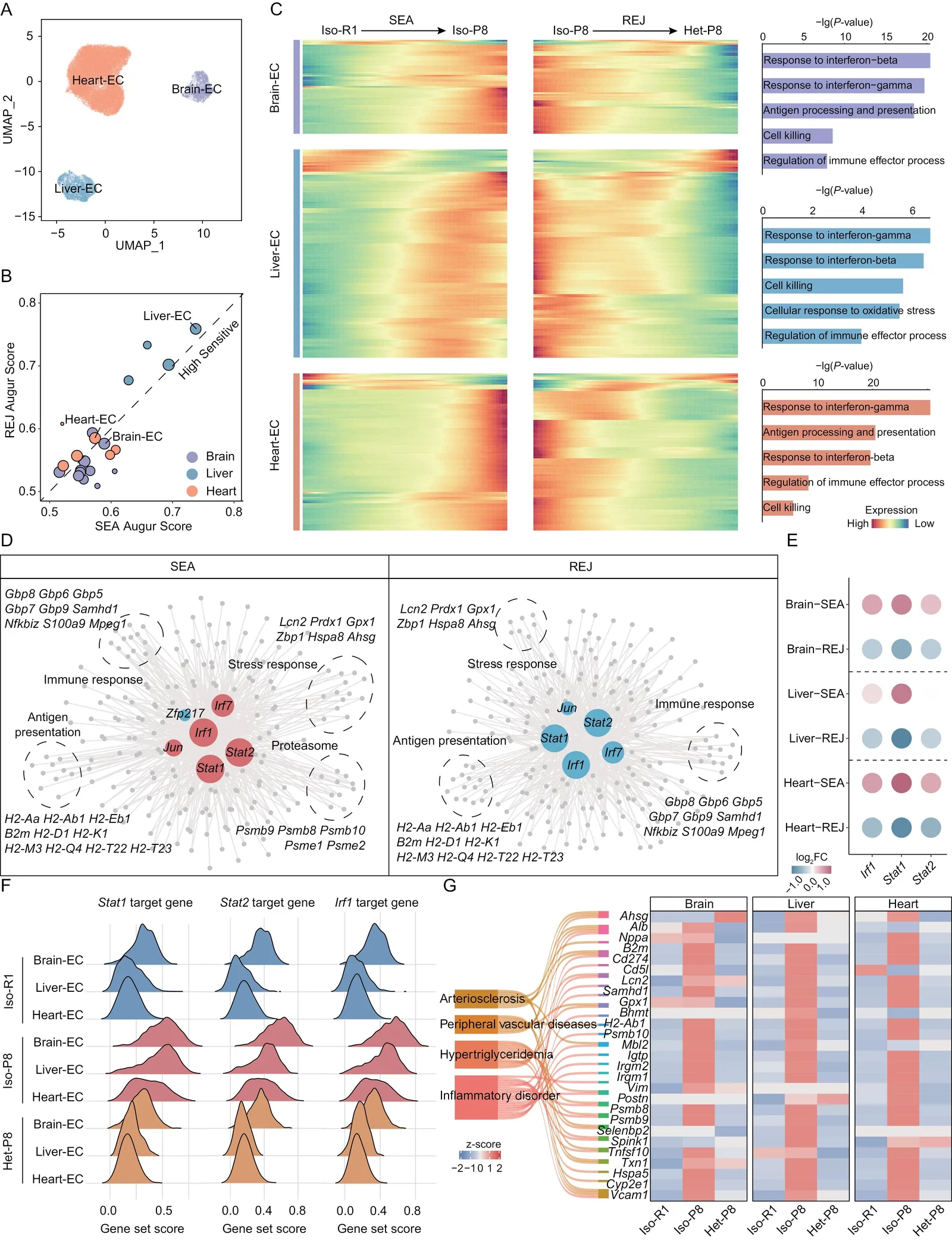
Attenuation of endothelial cell inflammation in the brain, liver, and heart by heterochronic parabiosis. (A) Uniform manifold approximation and projection (UMAP) plots showing endothelial cells across the brain, liver, and heart. (B) Dot plot showing the Augur scores of the brain, liver, and heart main cell types. (C) Heatmaps (left) showing pseudotime gene expression changes in endothelial cells across the brain, liver and heart. Bar plots (right) showing the enriched GO terms in the pseudotime DEGs. (D) Network plots showing the SEA and REJ endothelial cell TF regulatory networks. Red indicates upregulated transcriptional regulators. Blue represents downregulated transcriptional regulators. The size of each transcriptional regulator reflects the number of target genes. The gray symbols represent target genes. (E) Dot plot showing key regulator expression changes across the brain, liver, and heart in the SEA and REJ groups. (F) Ridge plots showing the changes in the expression of target genes regulated by key regulators. (G) Sankey plots (left) showing enriched vascular-related diseases. Heatmaps showing the changes in endothelial cell disease-related gene expression induced by heterochronic parabiosis.

ECs constitute the primary cell type lining blood vessel walls, regulating the exchange of substances between the blood and surrounding tissues and maintaining the barrier function of the vasculature [66]. By performing Augur analysis [67], a method for prioritizing the cell types most responsive to biological perturbations in single-cell data, as expected, we found that ECs are the most sensitive cell type to aging and rejuvenation via heterochronic parabiosis across all organs (Fig. 6A and 6B). To assess the molecular characteristics of ECs in the livers, brains, and hearts of mice subjected to heterochronic parabiosis, we conducted a pseudotime analysis and discovered that the changes in ECs across the three organs were remarkably similar [68] (Fig. 6C). In the SEA group, pathways related to the regulation of immune effector processes, cell killing, the response to interferon-beta and the response to interferon-gamma were activated in the ECs from the three organs. However, after heterochronic parabiosis, these pathways were suppressed. These results suggest that heterochronic parabiosis can abrogate the inflammatory state of ECs.

To further elucidate the gene regulatory network affected by heterochronic parabiosis in ECs, we utilized SCENIC to construct a transcriptional regulatory network. We found that heterochronic parabiosis upregulated the expression of genes related to the immune response (*Nfkbiz*, *S100a9*, and *Gbp8*), antigen presentation (*B2m*, *H2-Aa*, and *H2-D1*), and stress response (*Lcn2*, *Prdx1*, and *Gpx1*) driven by *Stat1*, *Stat2*, and *Irf1*. Heterochronic parabiosis also downregulated the expression of these TFs and their target genes (Fig. 6D). In heterochronic parabiosis, the expression of *Stat1*, *Stat2*, and *Irf1 in* ECs across the three organs was restored (Fig. 6E). Moreover, we used gene set scoring to assess the expression ratios of *Stat1*, *Stat2*, and *Irf1* and found that heterochronic parabiosis significantly downregulated the expression of these target genes (Fig. 6F).

EC health is crucial for maintaining vascular function and systemic homeostasis, and dysfunction of ECs can trigger the onset of various diseases [69]. Therefore, we performed disease pathway enrichment analysis on the genes whose expression was differentially expressed and found that heterochronic parabiosis downregulates several gene pathways associated with vascular diseases, such as arteriosclerosis, peripheral vascular disease, hypertriglyceridemia, and inflammatory disorders (Fig. 6G). *Vcam1* encodes the important cell adhesion molecule VCAM1, which promotes the adhesion of immune cells to the vessel wall and is one of the key markers of atherosclerosis. The upregulation of *Vcam1* increases the interaction between ECs and immune cells, promoting inflammation and plaque formation [70]. The upregulation of *B2m* is typically associated with chronic inflammatory responses [71]. In summary, heterochronic parabiosis can alleviate inflammation by downregulating the expression of inflammation-related TFs and various vascular disease risk genes.

### Heterochronic parabiosis reduces the expression levels of aging-related disease genes across multiple organs

**Figure 7.**
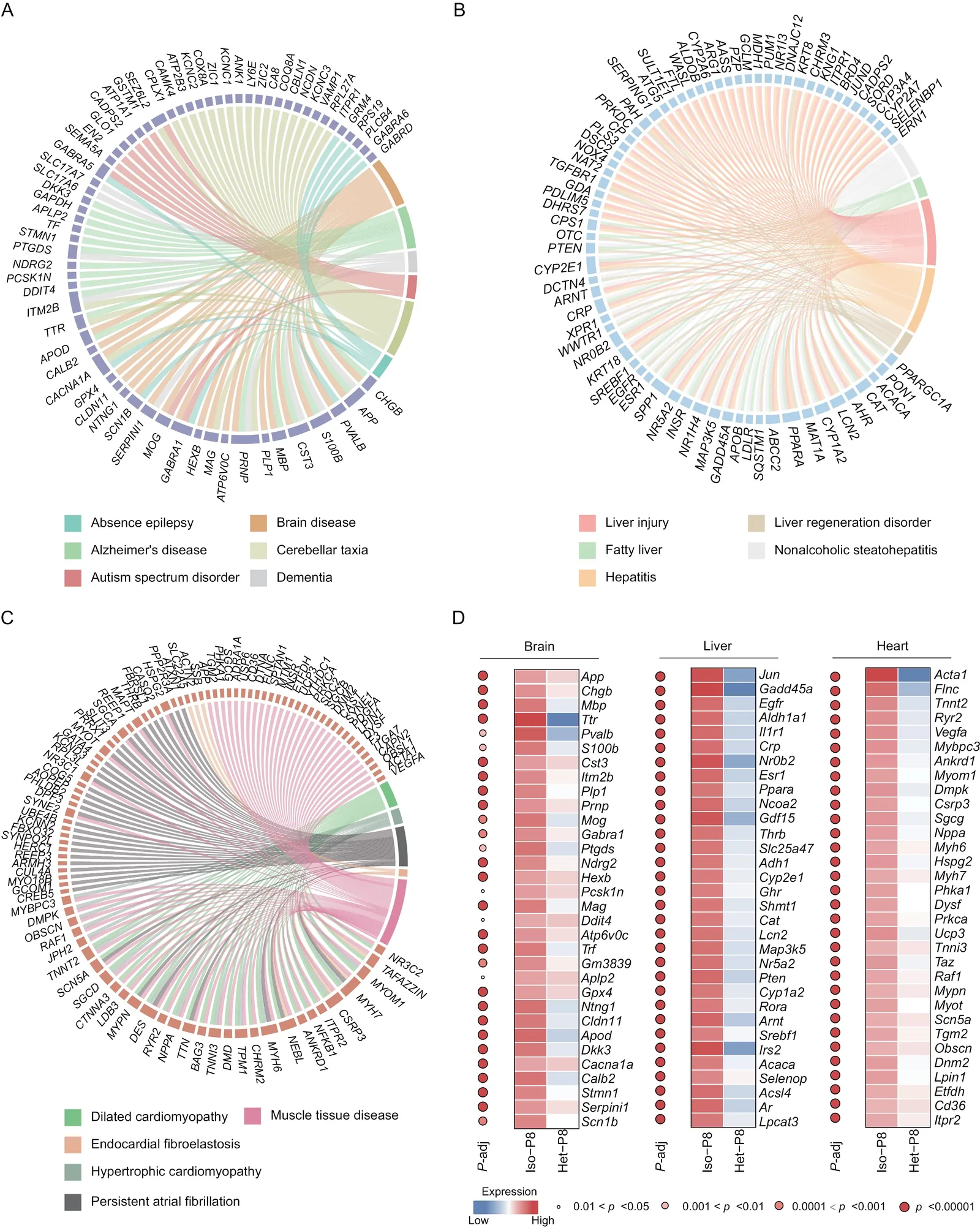
Decreased expression of genes involved in the aging phenotype and aging-related disease loci across the brain, liver, and heart by heterochronic parabiosis. (A) Chord diagram showing that REJ downregulated brain disease-related gene expression in neurons. (B) Chord diagram showing that REJ downregulated liver disease-related gene expression in hepatocytes. (C) Chord diagram showing that REJ downregulated heart disease-related gene expression in cardiomyocytes. (D) Heatmaps (right) showing the most significant improvement in the downregulated expression of genes associated with potential diseases in SAMP8 mice. Dot plots (left) showing *p*-adjusted values.

In the above analysis, we found that heterochronic parabiosis significantly ameliorates the degenerative aging features across multiple organs. Aging is recognized as a major risk factor for chronic diseases. We investigated the effect of heterochronic parabiosis on the expression of age-related disease risk genes. Therefore, we used the DisGeNET database [72] to annotate the downregulated genes in neurons (Fig. 7A), hepatocytes (Fig. 7B), and cardiomyocytes (Fig. 7C) in the REJ group, including genes involved in AD, dementia, autism spectrum disorder, fatty liver disease, nonalcoholic steatohepatitis, and hypertrophic cardiomyopathy and persistent atrial fibrillation. We selected the genes whose expression most significantly changed after heterochronic parabiosis in SAMP8 mice for display (Fig. 7D).

In the brain, *App* encodes the β-amyloid precursor protein, which is one of the core genes involved in AD. Aβ induces neurotoxicity, leading to synaptic dysfunction, neuronal apoptosis, and inflammatory responses [73]. *Prnp*, encoding a prion protein, may amplify the toxic effects of Aβ in AD, exacerbating synaptic dysfunction [74]. *Aplp2*, a member of the APP family, may cause neuronal apoptosis and worsen neural network degeneration when abnormally expressed [75]. *S100b* is often overexpressed in AD, triggering chronic inflammatory responses that further aggravate neuronal damage [76]. Heterochronic parabiosis can downregulate the genes involved in Aβ metabolism, neuroinflammation, and synaptic dysfunction, thereby counteracting age-related neurodegeneration.

In the liver, *Srebf1* regulates the expression of lipid metabolism-related genes and activates key enzymes (e.g. *Acaca*) involved in fatty acid and cholesterol synthesis. Overactivation of *Srebf1* leads to excessive production of fatty acids and triglycerides in the liver, directly contributing to abnormal fat accumulation in the liver, a hallmark of fatty liver disease [77]. *Crp*, an acute-phase protein [78], and *Il1r1*, a receptor for interleukin-1 [79], are typically elevated in fatty liver patients, promoting disease progression through chronic inflammation. Heterochronic parabiosis alleviates the key pathological basis of fatty liver disease by downregulating genes associated with aging-related lipid metabolism dysregulation and chronic inflammation.

In the heart, *Nppa* encodes atrial natriuretic peptide, a hormone secreted by cardiomyocytes [80]. The expression of *Nppa* is significantly elevated in cardiac fibrosis, reflecting myocardial stress, and serves as a key marker gene for cardiac remodeling [80]. *Cd36* is a fatty acid transporter protein. Its overexpression leads to excessive lipid accumulation, promoting myocardial lipotoxicity [81]. In addition, *Cd36* can bind to oxidized low-density lipoprotein, activating inflammatory signaling pathways, which further drive the progression of cardiac fibrosis. By regulating genes involved in lipid metabolism, inflammatory responses, ECM remodeling, and calcium signaling, heterochronic parabiosis effectively mitigates cardiac fibrosis and inflammation.

## Discussion

Aging is a complex, multifactorial process characterized by a progressive decline in function at the molecular, cellular, and tissue levels [1–3, 82]. This process is accompanied by disruptions in physiological homeostasis and a reduced capacity for regeneration, ultimately leading to decreased adaptability to external stressors and increased susceptibility to various diseases [3, 8]. Aging contributes to many chronic diseases, making it a key target for intervention. Among the various strategies aimed at delaying aging, heterochronic parabiosis has demonstrated considerable potential in mitigating aging-related diseases. In this study, we employed a SAMP8 accelerated aging mouse model to conduct a comprehensive analysis of gene expression changes at single-cell resolution, revealing how heterochronic parabiosis helps reverse functional decline and improve aging-related conditions across organs.

**Figure 8.**
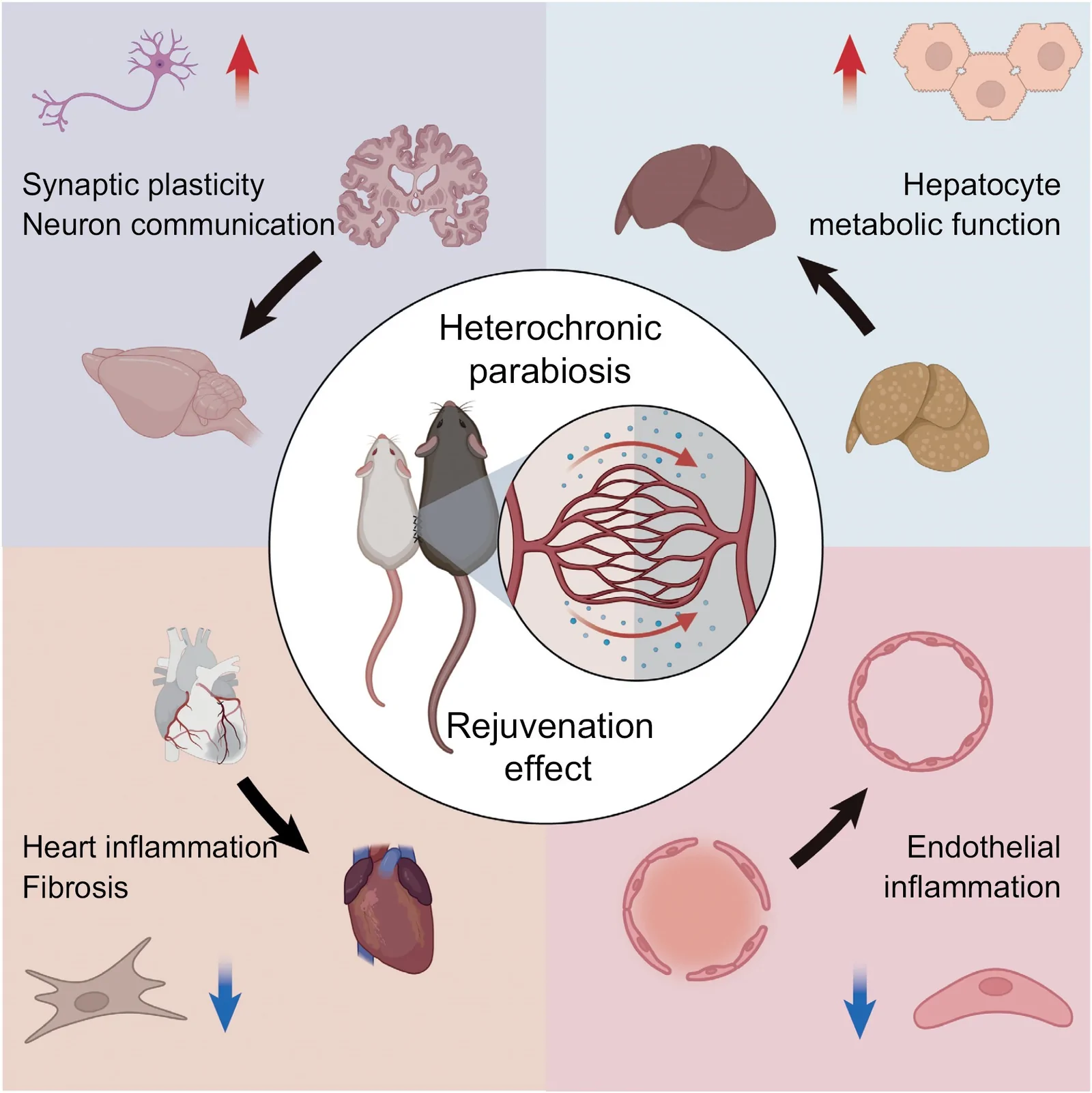
Summary schematic. It showing the regenerative effect of heterochronic parabiosis on aging-related disease in SAMP8 mice.

We initially focused on key pathway changes associated with aging. Aging is often accompanied by disruptions in cellular communication, persistent activation of inflammatory responses, metabolic dysregulation, impaired repair mechanisms, and a diminished capacity for tissue regeneration [1, 8]. Although SAMP8 is an accelerated aging mouse model, the key pathways and cellular mechanisms identified in this model are also relevant to normal aging, such as the decline in synaptic plasticity, the dysregulation of hepatocyte metabolic functions, and the chronic inflammatory state in cardiac multicellular systems. Our study shows that heterochronic parabiosis reverses gene expression changes across multiple cell types and organs, restoring function by enhancing synaptic plasticity, improving metabolism, and reducing inflammation and fibrosis (Fig. 8). Heterochronic parabiosis also exerts beneficial effects beyond gene expression changes. For example, in the liver, it upregulates *Acaa1b*, promoting fatty acid β-oxidation and reducing lipid accumulation [61, 62] (Fig. 4H). Heterochronic parabiosis significantly downregulated multiple disease-related genes. In the brain, it reduces the expression of genes involved in Aβ metabolism, whose abnormal activity contributes to neurotoxicity, synaptic dysfunction, AD, and other neurodegenerative diseases [73–76]. Overall, these findings further demonstrate the broad molecular effects of heterochronic parabiosis on aging and related diseases, providing critical scientific evidence for exploring therapeutic strategies to delay aging and address aging-related diseases.

One key finding is that heterochronic parabiosis significantly reduces inflammation in ECs. As the interface with the circulatory system, ECs act as both physical barriers and active regulators of the local microenvironment through cytokine secretion, blood flow regulation, and immune response involvement. As the first cell type to encounter circulatory factors from the young mouse during heterochronic parabiosis, ECs in our study exhibited the most sensitive responses to both aging and rejuvenation conditions (Fig. 6B). Thus, we hypothesize that ECs constitute a critical cell type that undergoes early functional changes during heterochronic parabiosis. By alleviating EC inflammation, heterochronic parabiosis may further restore local microenvironmental homeostasis, including reducing tissue inflammation, normalizing vascular function, and increasing intercellular communication, thereby laying the foundation for the recovery of organ function.

Heterochronic parabiosis, along with current mainstream antiaging approaches such as caloric restriction, senolytics, and physical exercise, can reshape the aged microenvironment and improve tissue homeostasis to varying degrees. Compared to these other interventions, heterochronic parabiosis induces more direct and rapid rejuvenation effects across multiple organs. However, it also faces ethical limitations that restrict its clinical application. In contrast, approaches such as caloric restriction, senolytics, and exercise are more ethically acceptable and safer, although they generally require longer intervention periods. Mechanistically, there are both overlaps and distinctions. For instance, extensive research shows that regular exercise and caloric restriction delay aging by restoring metabolic pathways [83, 84], consistent with our finding that heterochronic parabiosis improves hepatocyte metabolic function. Aging is a highly complex process, and future directions may focus on combining multiple interventions or developing cocktails of small molecules or bioactive factors from young blood to achieve synergistic antiaging effects.

Collectively, our analysis advances the understanding of the aging process and enhances the fundamental knowledge of the relationship between aging and aging-related diseases. This study provides valuable insights into the intrinsic connections between aging and disease and increases the understanding of the impact of heterochronic parabiosis on aging and its associated conditions. In addition, our study highlights the critical role of ECs in the processes of aging and heterochronic parabiosis. As cells are directly exposed to the circulatory system, ECs exhibit significant sensitivity during aging and may play an early and central role in the amelioration of aging-related functional impairments through heterochronic parabiosis. These findings offer new perspectives for developing therapeutic strategies targeting aging and aging-related diseases and provide potential targets for antiaging interventions aimed at achieving healthy aging.

## Research limitations

Although this study offers important insights, it has some limitations. First, the SAMP8 mouse model may not fully reflect the complexity of human aging. Second, single-cell omics cannot identify specific systemic factors in young mice, such as proteins, metabolites, or exosomes. Further research is needed to explore the mechanisms of heterochronic parabiosis and its potential in treating aging and related diseases.

## Methods

### Research ethics

All the mouse experiments were approved by the Institutional Animal Care and Use Committee of Peking University (SYXK2019-0032).

### Parabiosis

Following a previously reported method [25], an intraperitoneal injection of tribromoethanol (1.25%) was used for anesthesia, and carprofen (10 mg/kg) was administered for analgesia. For parabiosis surgery, mice were shaved and disinfected along the intended incision line (elbow to knee). A skin incision was made along the flank without damaging muscle and sutured using 4-0 polydioxanone (Jinhuan Medical, R413). The triceps and quadriceps of each pair were joined with two interrupted sutures, and the skin was closed. Mice recovered on a heated pad in the supine position. After surgery, pairs were housed individually and given daily subcutaneous injections of 0.25% bupivacaine in saline for 3 days. In the heterochronic group, a SAMP8 mouse was connected to a SAMR1 mouse for 4–5 weeks. The surgery had a 50%–60% success rate; failures were mainly due to anesthesia recovery issues, infection, poor wound healing, or incomplete vascular fusion. Postoperative care was essential for surgical success.

### Single-nucleus suspension preparation

Single-nucleus isolation was performed following a previously established protocol [85]. Frozen tissues were homogenized by pestle and filtered through a 100-μm cell strainer into a 1.5-mL tube, then transferred to a clean Dounce homogenizer containing 750 μL of lysis buffer (250 mM sucrose, 10 mg/mL BSA, 5 mM MgCl₂, 0.12 U/μL RNasin Plus, 0.12 U/μL RNasein, 1 × protease inhibitor, and 1% Igepal). Further homogenization was carried out using the tight pestle, followed by filtration through a 40-μm strainer. Nuclei were pelleted by centrifugation at 500 *g* for 5 min at 4°C and resuspended in a buffer containing 320 mM sucrose, 10 mg/mL BSA, 3 mM CaCl₂, 2 mM magnesium acetate, 0.1 mM EDTA, 10 mM Tris–HCl, 1 mM DTT, 1 × protease inhibitor, and 0.12 U/μL RNasein. After a second centrifugation, the nuclei pellet was resuspended in a cell resuspension buffer for library construction.

### snRNA-seq library preparation

Single-nucleus RNA sequencing libraries were generated using the DNBelab C Series Single-Cell Library Prep Set (MGI, 1000021082) following the manufacturer’s protocol. Key steps included droplet formation, emulsion breakage, bead recovery, reverse transcription, and cDNA amplification. Indexed libraries were constructed and quantified using the Qubit ssDNA Assay Kit (Thermo Fisher, Q10212). Sequencing was performed on a DNBSEQ-T1 or DNBSEQ-T10 platform (China National GeneBank, Shenzhen) using a 41-bp read for read 1 and a 100-bp read for read 2.

### snRNA-seq raw data processing

The sequencing data were processed according to previously described methods [86]. Bead barcodes and unique molecular identifiers (UMIs) were extracted from raw cDNA reads using the parse function in the PISA tool. Cell barcodes were located in bases 1–20 and UMIs in bases 21–30 of read 1. These sequences were added to the read ID lines to reformat the FASTQ files for downstream analysis. The reformatted reads were aligned to the mouse mm10 genome using STAR (v2.1). Gene annotations were added based on shared genes, and UMIs differing by a single base were corrected to preserve accurate cell barcodes and gene mapping. A gene expression matrix was then generated. Low-quality background cells were filtered out based on UMI count inflection points, identified using the barcodeRank function in the DropletUtils R package (v1.10).

### snRNA-seq data quality control and processing

The R package Seurat (version 4.3.0) was initially used to filter cells with fewer than 500 or more than 8000 detected features, as well as cells with fewer than 500 or more than 20,000 counts [87]. The R package DoubletFinder (version 2.0.3) was applied to identify and remove potential doublets. We then removed all the mitochondrial genes to eliminate their impact on single-nucleus sequencing [88]. We used the decontX (version 0.99.3) package to evaluate ambient RNA contamination in cells and removed those heavily affected by ambient RNA interference [89]. Finally, we manually removed some low-quality cells to ensure data quality.

### Integration, clustering, and identification of cell types

All high-quality samples were integrated using the Harmony (version 0.1.1) algorithm to correct for batch effects [90]. Next, 1500 highly variable genes were identified using the variance stabilizing transformation method to focus on genes whose expression significantly differed. After data integration, the “ScaleData” function was applied for scaling the data, followed by PCA using the “RunPCA” function, which is based on highly variable genes. Dimensionality reduction was then performed using the “RunUMAP” function. Cell clustering was refined using the “FindNeighbors” and “FindClusters” functions to identify distinct cell populations.

### Differential gene analysis

DEGs between the SEA and REJ groups were calculated using the Seurat package “FindMarkers” function with the parameters “min.pct = 0.1” and “min.cells.feature = 5.” Genes with |avg_log2FC| > 0.25 and adjusted *P*-value (P_val_adj) < 0.05 were identified as SEA DEGs. REJ genes were calculated using the same procedure. SEA DEGs that showed opposite changes in the REJ calculations with avg_log2FC > 0.1 were considered “reversed,” whereas those with avg_log2FC > 0.25 were classified as “rescued.”

### CV analysis

The CV was analyzed following previous work [35]. First, the CV was calculated between the Iso-R1 and Iso-P8 groups. Highly variable genes were selected for each cell type, and the absolute differences in expression for each gene across cells in the two groups were computed. The CV was then determined as the product of the standard deviation and the reciprocal of the mean. The CV between the Iso-R1 and Het-P8 groups was calculated following the same procedure. The difference between the two CV calculations was used to reflect the degree of change in each cell type due to heterochronic parabiosis.

### Pearson correlation analysis

The Pearson correlation coefficients were calculated using the R “cor()” function, and the results were scaled and visualized.

### Pathway enrichment analysis

GO enrichment analysis and GSEA were performed via R clusterProfiler [91] (version 4.2.2) and Metascape [92]. Cytoscape (version 3.8.0) software and R enrichplot (version 1.14.1) were used for visualization of the representative GO terms.

### Transcriptional regulatory network analysis

The Python implementation of SCENIC (pyscenic, version 0.12.1) was used to calculate the transcriptional regulatory network [40, 93]. The workflow included identifying coexpression modules, inferring regulons using cisTarget, and scoring regulon activity with AUCell.

### Cell-cell communication analysis

The R package CellChat (version 1.6.1) was used to infer intercellular communication networks from the snRNA-seq data [49, 50]. Only receptors and ligands expressed in more than 5% of cells in a specific cell type from the Iso-R1, Iso-P8, and Het-P8 mouse groups were included in the analysis. The average expression of each ligand‒receptor pair was calculated between each pair of cell types, and interactions with *P* values < 0.05 were retained as significant predictions of cell‒cell communication.

### Nonnegative matrix factorization

Consensus nonnegative matrix factorization (cNMF) was applied to infer transcriptional programs and define gene modules from snRNA-seq data [94]. The process involved decomposing the gene expression matrix into two lower-dimensional matrices, one representing the gene weights for each program and the other representing the contribution of each program across cells. cNMF was run iteratively to achieve a consensus clustering solution, ensuring the robustness and reproducibility of the identified transcriptional programs. On the basis of error and stability metrics, the optimal rank was determined to be *k *= 4, which was used for downstream analyses.

### Pseudotime analysis

The R package Monocle2 (version 2.22.0) was used to reconstruct the pseudotime trajectory [68]. Monocle uses the “detectGenes” function to filter genes with a minimum expression level of 0.1 and retains those expressed in at least 10 cells. Pseudotime analysis of DEGs was then performed with a *q*-value threshold of less than 0.01.

### Statistical analysis

All data were statistically analyzed using two-tailed *t*-tests and Wilcoxon tests to compare differences between groups. Statistical analyses were performed using R packages. A *P*-value < 0.05 was considered statistically significant.

## Supplementary Material

lnaf025_suppl_Supplementary_Figures_S1-S4

## Data Availability

This research generates no new code. All sequencing data are available in the China National GeneBank DataBase (CNGBdb) under accession number CNP0007085.
