# Supplementary material for: Heterochronic parabiosis alters the transcriptomic landscape to combat aging and aging-related diseases in aging-accelerated mice
Source: Life Med. 2025 Jul 1;4(6):lnaf025. doi: 10.1093/lifemedi/lnaf025 (PMC12732672; doi:10.1093/lifemedi/lnaf025)
Supplement: lnaf025_suppl_Supplementary_Figures_S1-S4 [file lnaf025_suppl_supplementary_figures_s1-s4.docx]

**Heterochronic parabiosis alters the transcriptomic landscape to combat aging and aging-related diseases in aging-accelerated mice**

Haochen Wang^1,#^, Wencong Lyu^1,#^, Yuzhe Sun^2,#^, Xinyi Jia^1,#^, Jinlong Bi^1^, Ran Wei^1^, Zhehao Du^1^, Fanju Meng^1^, Jianuo He^1^, Shiyi Wang^1^, Lijun Zhang^1^, Chao Nie^3,*^, Wei Tao^1,4,*^

^1^The MOE Key Laboratory of Cell Proliferation and Differentiation, School of Life Sciences, Peking University, Beijing 100871, China

^2^BGI Research, Beijing 102601, China

^3^ BGI Research, Shenzhen 518083, China

^4^Lead Contact

^#^These authors contributed equally to this work.

^*^These authors jointly supervised this work.

^*^Correspondence: niechao@genomics.cn (C.N.), weitao@pku.edu.cn (W.T.)

**Figure S1. Transcriptomic divergence across cell types in three organs.**

(A) Heatmap shows the Pearson correlation coefficients between different cell types in the brain. PN, Purkinje neuron; CGC, cerebellar granule cell; EN, excitatory neuron; IN, inhibitory neuron; MSN, medium spiny neuron; Micro, microglia; Astro, astrocyte; BGC, Bergmann glial cell; EPC, ependymocyte; EC, endothelial cell ; VLMC, vascular leptomeningeal myoendothelial cell; PC, pericyte; ABC, arachnoid barrier cell; CPC, choroid plexus epithelial cell; Olig, oligodendrocyte; OPC, oligodendrocyte precursor cell. (B) Heatmap shows the Pearson correlation coefficients between different cell types in the liver. Hep, hepatocyte; HSC, hepatic stellate cell; KC, Kupffer cell; EC, endothelial cell. (C) Heatmap shows the Pearson correlation coefficients between different cell types in the heart. CM, cardiomyocyte; Macro, macrophage; Fib, fibroblast; Mes, mesothelial cell; EC, endothelial cell; PC, pericyte.

**Figure S2. SynGO database GO enriched pathways of neuron SEA DEGs.**

1, postsynaptic specialization. 2, postsynaptic density. 3, postsynaptic density membrane. 4, integral component of postsynaptic density membrane. 5, postsynaptic membrane. 6, integral component of postsynaptic membrane. 7, postsynaptic ER. 8, postsynaptic cytosol. 9, presynaptic active zone membrane. 10, synaptic vesicle membrane. 11, extrinsic component of synaptic vesicle membrane.

**Figure S3. Neuron subtypes identification.**

CGC, cerebellar granule cell. EN_Epha7, Epha7 high expressed excitatory neuron. EN_Nrg3, *Nrg3* high expressed excitatory neuron. EN_Slc17a7, *Slc17a7* high expressed excitatory neuron, EN_Slc17a6, *Slc17a6* high expressed excitatory neuron. EN_Hs3st4, *Hs3st4* high expressed excitatory neuron. MSN_D1, medium spiny neuron with D1 dopamine receptor. MSN_D2, medium spiny neuron with D2 dopamine receptor. IN_Adarb2, *Adarb2* high expressed inhibitory neuron. IN_Sst, *Sst* high expressed inhibitory neuron. EN_Wipf3, *Wipf3* high expressed excitatory neuron. EN_Rxfp1, *Rxfp1* high expressed excitatory neuron. IN_Cnpy1, *Cnpy1* high expressed inhibitory neuron. EN_Ntng1, *Ntng1* high expressed excitatory neuron. PN, purkinje neuron.

**Figure S4. The rejuvenating effects of heterochronic parabiosis on multiple regions of the liver.**

(A) Network plot showing the enriched GO terms of downregulated (left) and upregulated (right) genes in SEA (Iso-P8/Iso-R1) group. (B) Gene markers of hepatocyte subtypes. pct.exp, percentage expression. avg.exp.scaled, average expression scaled. (C) Bridge plot showing the gene set scores of different gene modules. (D) Dot plot showing the PCA analysis of hepatocyte subtypes across different groups. (E) Bar plot showing the ratio of rescued genes in three hepatocyte subtypes. (F) Heatmaps showing the enriched GO terms of rescued genes. (G) Network plots showing the enriched GO terms of regulon targeted DEGs.
